# Prevention adopted by healthcare workers within their families in the Covid-19 pandemic

**DOI:** 10.1590/1980-220X-REEUSP-2021-0330

**Published:** 2022-01-07

**Authors:** Beatriz Rosana Gonçalves de Oliveira Toso, Bruna Regina Bratti Frank Terre, Ana Cristina de Oliveira e Silva, Elucir Gir, Juliano de Souza Caliari, Danielle Rosa Evangelista

**Affiliations:** 1 Universidade Estadual do Oeste do Paraná, Cascavel, PR, Brazil.; 2 Universidade Federal da Paraíba, Departamento de Enfermagem Clínica, João Pessoa, PB, Brazil.; 3 Universidade de São Paulo, Escola de Enfermagem de Ribeirão Preto, Ribeirão Preto, SP, Brazil.; 4 Instituto Federal do Sul de Minas Gerais, Passos, MG, Brazil.; 5 Universidade Federal do Tocantins, Palmas, TO, Brazil.

**Keywords:** Health Personnel, Covid-19, Disease Prevention, Personal de Salud, Covid-19, Prevención de Enfermedades, Pessoal de Saúde, Covid-19, Prevenção de Doenças

## Abstract

**Objective:**

To identify factors associated with the adoption of non-pharmacological preventive measures against covid-19 by healthcare workers within their families.

**Method:**

This is an analytical cross-sectional study carried out from October 1st to December 31st, 2020, with 11,513 healthcare workers in Brazil. Data collection through a virtual questionnaire on the platform Survey Monkey. To characterize the participants, descriptive statistical analysis was used with measures of absolute and relative frequency. Using inferential statistics, independent variables and outcome were compared, with hypothesis tests for association (chi-square, Fisher’s exact test), logistic regression, and Woe analysis. A significance level of 95% was used.

**Results:**

Most workers used measures such as hand hygiene, environmental sanitation, food hygiene, use of fabric masks, and physical distancing from family members. The association among variables was significant for the region, especially the South region, female sex, and nursing professionals.

**Conclusion:**

Healthcare workers adopt preventive measures against covid-19 within family life, especially the women and nursing professionals, with family isolation being the measure of greatest adherence.

## RESUMO

**Objetivo:**

Identificar fatores associados à adoção de medidas de prevenção não farmacológicas contra a covid-19 por profissionais de saúde no convívio familiar.

**Método:**

Estudo transversal analítico realizado de 01 de outubro a 31 de dezembro de 2020, com 11.513 profissionais de saúde do Brasil. Coleta de dados por meio de questionário virtual na plataforma *Survey Monkey*. Para caracterização dos participantes, utilizou-se análise estatística descritiva com medidas de frequência absoluta e relativa. Por meio de estatística inferencial, foram comparadas as variáveis independentes e desfecho, com testes de hipóteses para associação (qui-quadrado, teste exato de Fisher), regressão logística e análise Woe. Utilizou-se nível de significância de 95%.

**Resultados:**

A maioria dos profissionais utilizou medidas como higiene de mãos, limpeza de ambiente, higiene de alimentos, uso de máscaras de tecido e distanciamento físico dos familiares. A associação entre variáveis foi significante para região, em especial a Região Sul, sexo feminino e profissionais de enfermagem.

**Conclusão:**

Os profissionais de saúde adotam medidas de prevenção contra covid-19 no convívio familiar, destaque para o sexo feminino e profissionais de enfermagem, sendo o isolamento familiar a medida de maior adesão.

## RESUMEN

**Objetivo:**

Identificar factores asociados a la adopción de medidas de prevención no farmacológicas ante el Covid-19 por profesionales de salud en la convivencia familiar.

**Método:**

Estudio transversal analítico realizado de 01 de octubre a 31 de diciembre de 2020, con 11.513 profesionales de salud en Brasil. La recolección de datos fue realizada a través de cuestionario virtual en la plataforma Survey Monkey. Para caracterizar a los participantes, se utilizó el análisis estadístico descriptivo con medidas de frecuencia absoluta y relativa. Por medio de estadística inferencial, se compararon las variables independientes y resultado, con pruebas de hipótesis para asociación (chi cuadrado, prueba exacta de Fisher), regresión logística y análisis Woe.

**Resultados:**

la mayoría de los profesionales utilizó medidas como lavado de las manos, limpieza de ambiente, higienización de alimentos, utilización de mascarillas y aislamiento físico de los familiares. La asociación entre variables fue importante para la región, especialmente em la Región Sur, individuos del sexo femenino y profesionales de enfermería.

**Conclusión:**

Los profesionales de salud adoptan medidas de prevención ante Covid-19 en el convivio familiar, especialmente entre individuos del sexo femenino y profesionales de enfermería, por lo que el aislamiento familiar fue la medida de mayor adhesión.

## INTRODUCTION

The healthcare professionals’ work process comprises the care of human beings in all age groups and health needs^(1)^. Recently, this work process has undergone unexpected and rapid changes, due to the new pandemic that is plaguing the world as a whole. The accelerated increase in the contagion of people due to SARS-COV-2, which causes covid-19^(2)^, has been discussed. Furthermore, health professionals who are exposed to a high risk of infection with compromised mental health and, consequently, fear of disease transmission to their families, stand out^(3)^.

Nevertheless, the numbers of infected and dead people continue to increase globally. Until May 2021, 157,688,226 infected and 3,283,031 dead had been registered around the world as well as 15,145,879 infected and 421,316 dead in Brazil^(4)^, with the latter being one of the countries that admittedly dealt inadequately with the pandemic. In light of this reality, the health teams have gone through different experiences to manage this serious health condition, both in outpatient and hospital services.

These changes include the adoption of new techniques and procedures, new drugs, the use of personal protective equipment until then not required in daily work, with the exception of specific situations, such as N-95 or FFP2 masks, Face Shields, caps, goggles, gowns, overalls, gloves, and appropriate footwear. All this safety apparatus is still necessary in daily work to prevent contamination by the SARS-CoV-2 virus^(5)^.

It is known that transmission occurs through close and unprotected contact with secretions or excretions from an infected person, mainly through salivary droplets. Although not clearly explained, other bodily fluids such as blood, feces, vomiting, and urine can put the professional at risk^(6)^. Thus, prevention at work has been the most effective measure to prevent the illness.

Among the non-pharmacological measures to prevent contamination by covid-19, there is social distancing and/or isolation^(7)^. Unfortunately, healthcare workers cannot follow this recommendation, as their presence in their workplaces is critical to provide essential care to covid-19 patients. This results in the increased probability of work-related contamination in this new risk condition^(8)^.

In this context, the balance between work and family has become a challenge for healthcare workers, considering that contamination involves several aspects in the context of health work. Low-income countries recognize the contamination of health professionals as a professional deficiency^(3)^; therefore, prevention in the workplace becomes essential.

Prevention is understood at three basic levels: primary, secondary, and tertiary. According to this conception, primary prevention corresponds to general, educational measures to improve individuals’ resistance and general well-being so that they can withstand the aggressions of agents and the environment. The secondary one encompasses strategies for early detection of diseases, such as covid-19 screening tests. It also includes actions with individuals who are already sick, with confirmed diagnoses, so that they can be cured or remain functionally healthy, through preventive clinical practices and health education. Finally, the tertiary one concerns the care of individuals with sequelae of illnesses or accidents, aiming at recovery or maintenance in functional balance^(9)^. In this respect, the level of prevention against covid-19 adopted by healthcare workers in their work environment is the primary one.

In this article, we focus on the non-pharmacological prevention measures adopted by healthcare workers who worked in direct assistance to the individual in different health care scenarios, regardless of the diagnosis of covid-19, to avoid the contamination of people in their family environment, as well as on the factors associated with the adoption of such measures. Thus, the objective of the study was to identify factors associated with the adoption of non-pharmacological preventive measures against covid-19 by healthcare workers in their family context.

## METHOD

### Type of Study

Analytical cross-sectional study, online survey type, held from October 1st to December 31st, 2020 throughout the Brazilian territory. This study followed the recommendations of the Strengthening the Reporting of Observational Studies in Epidemiology (STROBE) and was guided by the Checklist for Reporting Results of Internet E-Surveys (CHERRIES).

This study is part of the Multicenter Project related to the effects and consequences of the covid-19 pandemic among healthcare workers in Brazil.

### Population and Sample

The study included 12,086 healthcare workers who provided direct patient care, regardless of the suspected or confirmed diagnosis of covid-19, in public and/or private health services, at least in the last six months prior to the beginning of collection, and who declared they were living with family members during the pandemic. For this part of the research, 11,513 healthcare workers were eligible, considering that they responded to the variables related to non-pharmacological preventive measures in family life.

However, in a study with this scope, some questions, such as age, for example, sometimes remain unanswered, reducing the number of respondents in some items. Thus, participants with missing data in the age variable were excluded from the database when performing the analysis of this variable with the outcome. Moreover, it is important to highlight as a research bias that, as this is a study carried out online, healthcare workers who did not meet the eligibility criteria may have responded to the questionnaire.

### Data Collection

For the data collection stage, carried out from October 2 to December 31, 2020, a previously trained team recruited individuals through digital media (*Whatsapp*, *Facebook, Instagram*), by sending a link for access to virtual documents: the Free and Informed Consent Form (FICF) and the survey form. The decision to use a virtual questionnaire was mainly to allow the participation of professionals from all regions of Brazil, and to consider the recommendation for non- pharmacological measures to prevent covid-19, such as social distancing.

The filled instruments were hosted in the software Survey Monkey, which allowed a single submission of the form per IP (Internet Protocol), aiming at the safety of the information collected.

### Data Collection Instrument

The data collection instrument was built and validated by fifteen experts on the topic of infectious-contagious diseases or health care-related infection control. The instrument consists of multiple-choice questions, some of which being mandatory to proceed, divided into demographic and individual information such as professional category, type of care provided, variables related to preventive measures adopted in family life and on the diagnosis of covid-19 among healthcare workers.

### Data Analysis and Treatment

Data were collected through the platform Survey Monkey, exported and analyzed in the statistics software R, version 4.0.4. To characterize the participants, descriptive statistical analysis was used with measures of absolute and relative frequency. The dependent variable considered was the adoption of preventive measures within family life, while the following were considered as independent variables: sex, age group, region, professional category, marital status, living with children under 12 in the home environment, living with older people and people of risk groups in the home environment, diagnosis of covid-19, and care provided in a field hospital for covid-19.

In this study, the preventive measures against covid-19 in the family environment that were considered were the non- pharmacological measures of prevention against covid-19 defined by the Centers for Disease Control and Prevention (CDC), which are hand hygiene, use of masks, family distancing, among others^(10)^. Thus, workers who adopted preventive measures within family life, that is, who answered “yes” to the dependent variable, were those who indicated at least three alternatives: hand hygiene, use of masks, and physical distance from family members.

To compare the independent variables and the outcome variable, hypothesis tests for association (chi-square and Fisher’s exact test) were used, using inferential statistics. Also, to provide evidence on factors that are related to the study outcomes, logistic regression methods were considered. The confidence level adopted in all analyses was 95%. Predictive variables were considered using the WOE (Weight of Evidence)^(11)^.

The WOE corresponds to the weight of evidence of a set of explanatory variables intended to explain the occurrence of an outcome, characterized by a dichotomous variable Y. It is given as a function of a statistical model, and the model adjustment provides the measure of information value (IV), which allows showing the strength of each explanatory variable for the outcome. Ranking values indicate the following: if *IV*< 0.02: not predictive; if 0.02 ≤ *IV*< 0.1: weak; if 0.1 ≤ *IV*< 0.3: strong (or average); if *IV*≥ 0.3: very strong (suspicious)^(11)^.

For the variables considered significant, a logistic regression model between the adoption of prevention measures and these variables was adjusted to estimate the chance of adopting prevention measures considering the influence of these significant variables.

### Ethical Aspects

The project was approved by the Research Ethics Committee (CEP) under opinion number 4.258.366, in 2020. All ethical aspects were considered for its performance according to Resolutions no. 466/2012 and no. 510/2016. The FICF was signed online by the participants before they filled out the instrument.

## RESULTS

The study included 11,513 healthcare workers from all regions of Brazil, most of them belonging to the Northeast Region, 3514 (30.5%), followed by the Southeast, 3316 (28.8%), Central West, 2002 (17.38%), North, 1710 (14.8%), and South, 971 (8.4%) regions. Regarding the professional category, most were nursing professionals, 8685 (75.4%), followed by physicians, 1152 (10%), physiotherapists, 647 (5.6%), professionals from the category others, 532 (4.6%, dentists, 233 (2.02%), psychologists, 174 (1.41%), speech therapists, 54 (0.4%), and occupational therapists, 36 (0.3%). Regarding sex, there was a predominance of women, 9,313 (80.9%), aged between 31 and 60 years, 7,037 (61.12%), and married or in a common-law marriage, 6,057 (52.6%).

Regarding the healthcare workers’ family life during the covid-19 pandemic, most reported having spent a period isolated from the family, 7,519 (65.3%).

Among the preventive measures against covid-19 used in the home environment, hand hygiene had the highest frequency, 11,025 (95.8%), followed by environmental sanitation, 10,107 (87.8%), food hygiene, 8,526 (74.1%), use of fabric masks, 6,325 (54.9%), and use of N-95 mask, 1685 (14.6%). The physical distance from family members was present for 5,283 (45.9%) (Table 1).

**Table 1.** Frequency of covid-19 prevention measures adopted by healthcare workers within family life – Brazil, 2020. (n = 11,513).

Variables	n (%)
**Hand hygiene**	
Yes	11,025 (95.8)
No	488 (4.2)
**Environmental sanitation**	
Yes	10,107 (87.8)
No	1,406 (12.2)
**Food hygiene**	
Yes	8,526 (74.1)
No	2,987 (25.9)
**Use of fabric masks**	
Yes	6,325 (54.9)
No	5,188 (45.1)
**Use of N-95 masks**	
Yes	1,685 (14.6)
No	9,828 (85.4)
**Physical distancing from family members**	
Yes	5,283 (45.9)
No	6,230 (54.1)
**Separation of household items**	
Yes	2,124 (18.4)
No	9,389 (81.6)
**Home isolation**	
Yes	2,092 (18.2)
No	9,421 (81.5)
**Moving from home**	
Yes	544 (4.7)
No	10,969 (95.3)
**Total**	**11,513 (100.0)**

Source: Research database.

The association between demographic and individual variables with the adoption of covid-19 prevention measures in family life by healthcare workers was significant for the region (p = 0.036), sex (p < 0.001), professional category (p = 0.018), presence of older people or people at risk for Covid-19 in the family (p = 0.004) (Table 2). The other variables analyzed were not considered significant, namely: marital status (p = 0.154), age group (p = 0.123), diagnosis of covid-19 (p = 0.921), existence of children under 12 years living with the respondent (p = 0.780), and service provision in a field hospital (p = 0.603).

**Table 2.** Results of the test of association between demographic and individual variables with the use of preventive measures against covid-19 in family life by healthcare workers and results of the odds ratio adjusted by the logistic regression model – Brazil, 2020.

Variables	Adoption of measures to prevent Covid-19		p–value related to the category	Adjusted OR	95% CI OR	
	Yes	No			LL	UL
	n (%)	n (%)				
**Region** ^(1)^						
Northeast	3514 (30.5)	73 (23.2)	–	–	–	–
North	1710 (14.9)	36 (11.5)	0.831	–	–	–
Central west	2002 (17.4)	71 (22.6)	**0.017**	0.665	0.476	0.930
Southwest	3316 (28.8)	89 (28.3)	0.313	–	–	–
South	971 (8.4)	45 (14.3)	0.000*	0.487	0.332	0.715
**Sex** ^(1)^						
Male	2200 (19.1)	108 (34.4)	–	–	–	–
Female	9313 (80.9)	206 (65.6)	0.000*	1.887	1.467	2.427
**Marital status** ^(2)^						
Single/Divorced	5387 (46.8)	135 (43.0)	–	–	–	–
Married/common-law marriage	6057 (52.6)	177 (56.4)	–	–	–	–
Widow	69(0.6)	2 (0.6)	–	–	–	–
**Professional category** ^(1)^						
Physician	1152 (10.0)	78 (24.8)	–	–	–	–
Nurse	5627 (48.9)	142 (45.2)	0.000*	2.100	1.559	2.830
Nursing technician	2875 (25.0)	57 (18.2)	0.000*	2.614	1.824	3.747
Nursing assistant	183 (1.6)	5 (1.6)	0.148	1.981	0.784	5.006
Physiotherapy	647 (5.6)	12 (3.8)	0.000*	3.018	1.624	5.607
Psychologist	174 (1.5)	5 (1.6)	0.167	–	0.761	4.834
Speech therapist	54 (0.5)	1 (0.3)	0.268	–	0.420	22.844
Occupational therapist	36 (0.3)	1 (0.3)	0.593	–	0.232	12.882
Dentist	233 (2.0)	3 (1.0)	**0.013**	4.407	1.374	14.131
Other	532 (4.6)	10 (3.2)	**0.002**	2.876	1.470	5.626
**Age group** ^(2)^						
18 to 30	4114 (77.09)	99 (83.2)	–	–	–	–
31 to 60	1220 (22.86)	20 (16.8)	–	–	–	–
61 or more	2 (0.05)	0 (0.0)	–	–	–	–
**Diagnosis of Covid-19** ^(1)^						
Yes	7837 (68.1)	194 (61.8)	–	–	–	–
No	3676 (31.9)	120 (38.2)	–	–	–	–
**Lives with older people or those at risk for covid-19** ^(1)^						
Yes	3875 (33.7)	62 (19.7)	0.000*	1.777	1.337	2.362
No	7638 (66.3)	252 (80.3)	–	–	–	–
**Lives with children under 12 years old** ^(1)^						
Yes	4108 (35.7)	98 (31.2)	–	–	–	–
No	7405 (64.3)	216 (68.8)	–	–	–	–
**Provided assistance in a field hospital for covid-19** ^(1)^						
Yes	3418 (29.7)	82 (26.1)	–	–	–	–
No	8095 (70.3)	232 (73.9)	–	–	–	–

*p-value less than 0.01.^(1)^ Chi-Square Test;^(2)^ Fisher’s Exact. LL – Lower Limit; UL – Upper Limit. Source: Research database.

According to the logistic regression model, the results of the odds ratios for the variables that had significant OR measures are shown in Table 2.

The results show that professionals from the North and Southeast regions have the same chances of adopting prevention measures when compared to professionals from the Northeast Region. A professional from the Central West Region (OR = 0.665; CI: 0.476–0.930; p = 0.017) is 33.5% less likely to adopt covid-19 preventive measures compared to a professional from the Northeast Region.

In the association with the variable sex, it was found that a female professional (OR = 1.887; CI: 1.467–2.427; p < 0.01) is 88.7% more likely to adopt covid-19 prevention measures when compared to a male professional. Also following this analysis, the healthcare worker who has older people or people in a risk group at home (OR = 1.777; CI: 1.337–2.362; p < 0.01) are 77.7% more likely to adopt covid-19 prevention measures when compared to those who do not have them in their household.

Regarding the profession, professionals in the fields of psychology, speech therapy and occupational therapy, and nursing assistants have the same chances of adopting measures to prevent covid-19 when compared to physicians. However, the nurse (OR = 2.100; CI: 1.559–2.830; p < 0.01) is 110% more likely to adopt covid-19 preventive measures when compared to a physician. The nursing technician (OR = 2.614; CI: 1.824–3.747; p < 0.01) is 161.4% more likely to adopt covid-19 preventive measures when compared to a physician. The physiotherapist (OR = 3.018; CI: 1.624–5.607; p < 0.01) is 201.8% more likely to adopt preventive measures, and the dentist (OR = 4.407; CI: 1.374–14.131; p = 0.013) is 340.7% more likely to adopt preventive measures, when compared with a physician.

According to an analysis using the Woe method, the results show that the two variables most strongly associated with the outcome “Adoption of preventive measures within family life” are, in this order, sex (higher weight of categories: female) and professional category (higher weight of categories: nursing professional and physician, in this order). Other variables that individually have a weak relationship to explain the outcome were detected: “There are older people or people from the risk group in the family environment” (higher weight of the categories: yes), “Region of the country where person lives” (higher weight of regions: South), and “Marital status” (higher weight of the categories: married and single, in this order).

**Figure 1.**
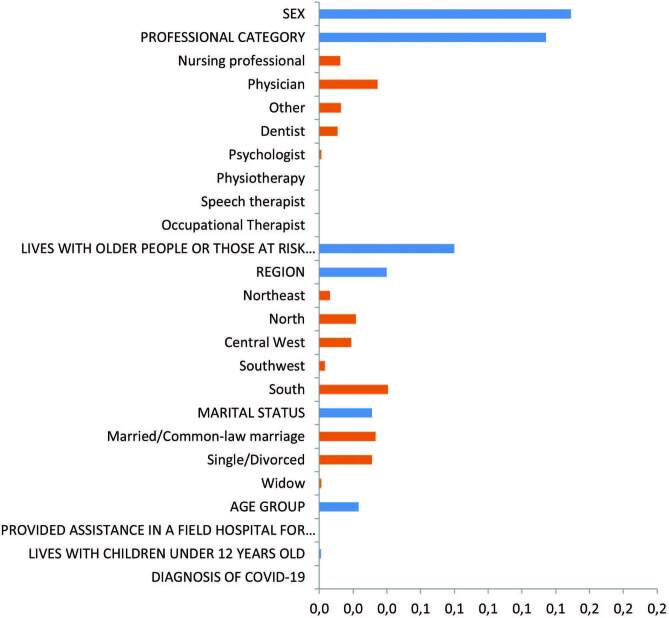
Information values obtained by the WoE (Weight of Evidence) method to explain the outcome “Adoption of preventive measures in family life”. Brazil, 2020.

In addition, the variables “Provided care in a field hospital”, “There are children under 12 in the family”, “Age group” and “Diagnosis of covid-19” did not provide evidence of being statistically associated with the outcome, pointed out by the chi-square tests performed for these variables (Figure1).

## DISCUSSION

This study evaluated the adoption of non-pharmacological measures to prevent covid-19 by healthcare workers within family life, considering aspects related to family isolation, the presence of older people and/or children under twelve years in the family environment and frequency of the main measures adopted in the family life.

Regarding the participants’ characterization, the population consists mostly of young women, nursing professionals, married or in a common-law marriage. The professional profile data converge with the national reality, in which most workers in the health area are women (83.8%), as reported in the bulletin *Emprego em Pauta (Employment on the Agenda),* of the Inter-Union Department of Statistics and Socio-Economic Studies (Dieese)^(12)^, which presents the socioeconomic characteristics of health professionals in Brazil.

In this area, the inequality between physicians and nursing professionals (nurses, nursing technicians and assistants) stands out. Among physicians, most of them are white men. In the nursing category, among nurses, there is a higher prevalence of white women. Among technical nursing professionals, in their turn, they are mostly women and black^(12)^. In addition, the number of nursing professionals is the highest in the health area, currently being of approximately 70% of professionals (17% nurses, 53% of nursing assistants and technicians)^(13)^ in Brazil.

The main problem affecting professionals involved in the care of symptomatic patients or those diagnosed with covid-19 infection is the risk of infection due to the high level of exposure to the virus^(14)^. The risk of contamination is greater among nursing professionals because they are directly involved in patient care, and due to the exhaustion and stress resulting from double and long working hours^(15)^.

Corroborating the authors, a study carried out in the state of Amapá analyzed the profile of healthcare workers affected by covid-19 between March and May 2020 and demonstrated that the nursing category was the most affected (42% secondary education and 16% higher education level), with women representing 64.5% of those affected and the most relevant age group ranging from 31–45 years with 56% of cases, showing a characterization of professionals similar to this study^(16)^.

In this regard, given the increased risk of contamination of healthcare workers and the consequent transmission of the virus to their families and colleagues or even to the community in which they live, it is important to adopt clear strategies to deal with this situation in family life, such as the physical distancing from family members reported by 45.9% of study participants.

In line with this panorama, an article dealing with monitoring approaches for healthcare workers during the covid-19 pandemic emphasizes that physical distancing should be encouraged, both for contact with colleagues, during meetings, meals, and at work offices^(17)^, and with family members, as in the findings of this study.

Still on this research trail, results of a survey in a study carried out to analyze the experiences of healthcare workers in dealing with the coronavirus pandemic (covid-19), with approximately 1,036 health professionals, showed that 70% were women, 52% belonged to the age group of 26–34 years, 50% were nurses, 33.7% were physicians, 97.7% believed that they should prevent infection among healthcare workers and provide safety to family members, almost 94% believed that appropriate personal protective equipment (PPE) increased their availability to show up to work^(18)^.

Among the prevention measures adopted by the healthcare workers interviewed are hand washing, use of masks, and environmental and food sanitation, measures that can influence the reduction of the risk of contagion. Such measures corroborate the prevention and control actions recommended by the Brazilian Health Regulatory Agency (ANVISA)^(5)^, namely: hand washing with soap and water and their sanitation with 70% alcohol, use of surgical masks and other personal protection equipment intended for the care of suspected or confirmed cases of covid-19.

In contrast, in a study carried out on prevention in Africa, with limited supply of PPE, even those of low cost, such as face masks and water sources for washing hands, the situation can be challenging, leading to high contamination among healthcare workers and their families, and can be worsened by the number of limited intensive care beds and the difficulties in transporting sick healthcare workers from rural to urban centers^(19)^.

In Brazil, this scenario is no different, as the constant lack of PPE, such as gloves, traditional and specific masks, as well as other protective equipment, added to the intense work hours due to insufficient human resources, ends up generating stress, fear, and insecurity in healthcare workers^(20)^.

Similar to the behavior adopted by the respondents in this study, American health professionals, who participated in a survey aiming to assess the factors that contributed to covid-19 infection and psychological distress during the pandemic, in the US, they reported that most health workers took precautions to protect the individuals they lived with, including all necessary precautions at home (56.96%), moving to a different residence temporarily (12.09%), or sending cohabitants out of home (7.27%)^(21)^. In this regard, it is evident that the fear of contamination and the consequent transmission of the virus to colleagues, family members, and the community can be associated with the adoption of preventive measures by professionals within family life.

A study carried out in France to bring up-to-date information about the potential risks to mental health associated with the exposure of healthcare workers to the covid-19 pandemic indicated a change in social and family daily life, added to concerns about their own health, fear of taking the infection to family members or others, the possibility of social isolation, feelings of uncertainty and social stigmatization, and work overload^(22)^.

However, problems such as physical fatigue, psychological stress, insufficiency and/or negligence regarding the protection and health care measures of professionals do not affect the different categories in the same way, and attention to the specificities of each one is required^(23)^.

The studies mentioned corroborate the data from this research, demonstrating that the concern of healthcare workers is real, especially of female nursing professionals, who have a family member to protect, with measures to prevent contamination by SARS-CoV-2, such as physical distance from family members, hand washing, environmental and food sanitation, and wearing a mask.

This study has as a limitation the cross-sectional approach, which shows the association among the variables, but does not analyze the meanings of the professionals’ responses, such as the reason why most participants do not adopt social isolation, which would require an associated qualitative study. The online form of data collection shall be seen as a limiting factor, as it creates some distance between researchers and research participants. On the other hand, given the health situation resulting from the pandemic, the online collection allowed the performance of this study, with no harm to the quality of information.

As implications for professional practices, knowing these data can provide subsidies to health professionals so that they do not feel alone in their decisions to adopt preventive measures, since they are efficient and necessary to avoid the contamination of people whom they live with, although currently they clearly are not a government policy in the country.

## CONCLUSION

The data found indicate that factors related to the adoption of preventive measures by health professionals are mainly related to sex, especially for women, and the nursing profession. Professionals have adopted preventive measures against covid-19, avoiding spreading this disease to their families, through simple measures such as proper hygiene and the use of masks, but also through isolation. These measures can lead to other consequences for the worker’s health, such as mental problems arising from the situation experienced, due to the fear of contaminating their families and community.
